# Do Phosphate and Cytokinin Interact to Regulate Strigolactone Biosynthesis or Act Independently?

**DOI:** 10.3389/fpls.2020.00438

**Published:** 2020-05-20

**Authors:** Kaori Yoneyama, Xiaonan Xie, Takahito Nomura, Koichi Yoneyama

**Affiliations:** ^1^Graduate School of Agriculture, Ehime University, Matsuyama, Japan; ^2^PRESTO, Japan Science and Technology, Kawaguchi, Japan; ^3^Center for Bioscience Research and Education, Utsunomiya University, Utsunomiya, Japan; ^4^Women’s Future Development Center, Ehime University, Matsuyama, Japan

**Keywords:** strigolactone, cytokinin, phosphate deficiency, biosynthesis, rice

## Abstract

Strigolactones (SLs) are essential host recognition signals for both root-parasitic plants and arbuscular mycorrhizal (AM) fungi in the rhizosphere, and *in planta* SLs or their metabolites function as a novel class of plant hormones that regulate various aspects of plant growth through crosstalk with other hormones. Although nutrient availability is one of the important factors influencing SL production and exudation, and phosphate (Pi) deficiency significantly promotes SL production and exudation in host plants of AM fungi, how nutrient availability modulates SL production and exudation remains elusive. Cytokinin (CK), a canonical plant hormone, has extensively been studied as a shoot branching promoter and its biosynthesis is also influenced by mineral nutrients, especially nitrate, indicating that CK might be another key factor that affect SL production and exudation. In the present study, we show that CKs (*t*-zeatin, benzyladenine, kinetin, and CPPU) applied to hydroponic culture media significantly suppressed the SL levels in both the root exudates and the root tissues of rice plants grown under Pi deficiency. In a split-root system, CK suppressed SL production locally, while Pi affected SL production systemically, suggesting that Pi and CK act on SL production independently in rice plants.

## Introduction

Strigolactones (SLs) exuded from plant roots are essential host recognition signals for both root-parasitic plants (Cook et al., 1966) and arbuscular mycorrhizal (AM) fungi (Akiyama et al., 2005) in the rhizosphere. Root-parasitic plants deprive water and nutrients from their host plants, causing severe damages to the host plants. Thus, most of the root-parasitic plants are generally regarded as agricultural pests. Unfortunately, economical and effective control methods for the weedy root parasites have not yet been established. By contrast, AM fungi stay in the cortical tissues of host roots and supply mineral nutrients, mainly phosphate (Pi), to the host plants. More than 80% of land plants form symbiotic relationships with AM fungi. The interactions between root-parasitic plants and symbiotic AM fungi with their host plants have been reported to be influenced by nutrient availability in the soil; fertilizations mitigate the damages of root-parasitic weeds growing on nutrient-poor soils but inhibit AM colonization. Since SL production and exudation are regulated by plant nutrients including Pi and nitrogen (N) (Yoneyama et al., 2007a, b), nutrient availability in soils is regarded as one of the important factors controlling the parasitism of root-parasitic weeds and AM symbiosis. Phosphate regulation of AM symbiosis acts in part through SL, but not exclusively. In addition to SL, other mechanisms also regulate Pi response of AM colonization (Breuillin et al., 2010; Balzergue et al., 2011; Foo et al., 2013).

In plants, SLs or their metabolites play pivotal roles in various aspects of plant growth as a novel class of plant hormones through crosstalk with other hormones. SL biosynthesis mutants display excessive shoot branching, and SL application rescues this phenotype (Gomez-Roldan et al., 2008; Umehara et al., 2008). Subsequent studies with SL mutants and on the effects of application of SL revealed that SLs are involved in the regulation of root architecture (Kapulnik et al., 2011; Ruyter-Spira et al., 2011; Arite et al., 2012), secondary growth (Agusti et al., 2011), leaf senescence (Snowden et al., 2005; Yamada et al., 2014; Ueda and Kusaba, 2015), and response to abiotic stresses (Ha et al., 2014). Shoot branching is also affected by nutrient availability (Umehara et al., 2010; de Jong et al., 2014; Shindo et al., 2018). SLs also play a role in nodulation (Soto et al., 2010; Foo and Davies, 2011; McAdam et al., 2017). When plants are grown under nutrient-rich conditions, SL production and exudation are reduced and shoot branching is promoted. On the other hand, Pi and N starvation increases SL production and results in the inhibition of shoot branching.

**FIGURE 1:**
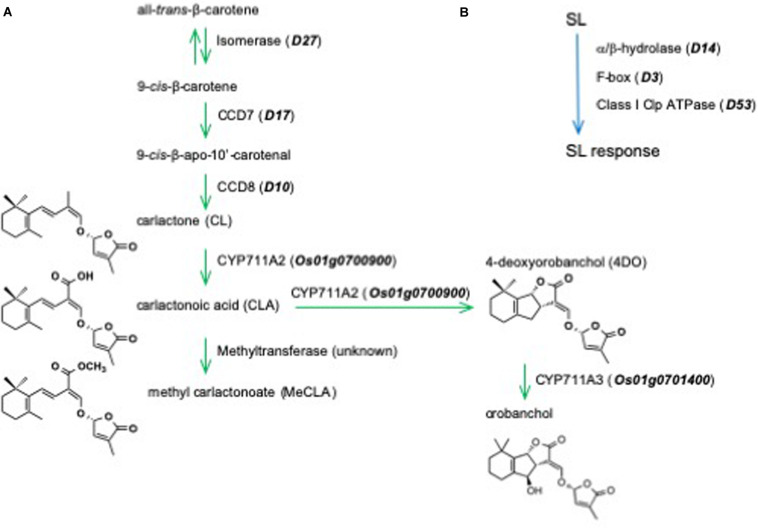
**(A)** The biosynthetic pathway and **(B)** perception and signaling in the shoot branching inhibitory activity of strigolactones in rice.

The core pathway of SL biosynthesis starts from all-*trans*-β-carotene, which is converted into 9-*cis*-β-carotene by an isomerase, D27 (Figure 1A). Then, 9-*cis*-β-carotene is converted by carotenoid cleavage dioxygenase (CCD) 7 and CCD8 into carlactone (CL), the first SL backbone molecule (Alder et al., 2012) and a precursor for SLs (Seto et al., 2014). In rice plants, CL is then converted into 4-deoxyorobanchol (4DO) *via* carlactonoic acid by CYP711A2 (Zhang et al., 2014; Yoneyama et al., 2018). Another cytochrome P450, CYP711A3, converts 4DO into orobanchol (Zhang et al., 2014; Yoneyama et al., 2018).

The SL receptor D14 is unique among plant hormone receptors, which belong to the superfamily of α/β-hydrolases and has the ability to bind and hydrolyze SLs. The F-BOX protein D3 and the transcriptional repressor D53 are also involved in SL perception and signaling (Figure 1B). The mechanisms of SL perception and signaling are still open for discussion (Waters et al., 2017; Shabek et al., 2018; Marzec and Brewer, 2019; Seto et al., 2019; Blázquez et al., 2020).

Biosynthesis of SLs seems to be regulated through crosstalk with other plant hormones. Before the discovery of the new function of SLs as a branching inhibitor, cytokinin (CK) and auxin had been extensively examined as factors that regulate shoot branching. Direct application of CK to the axillary bud promoted shoot branching (Dun et al., 2012). It was suggested that auxin derived from the shoot apex suppresses the local CK biosynthesis and decreases the CK levels, resulting in the inhibition of shoot branching. Our previous studies demonstrated that indole-3-acetic acid (IAA) applied to the shoot apices in sorghum increased the root contents of 5-deoxystrigol (5DS), one of the major SLs produced by sorghum plants (Yoneyama et al., 2015). It was shown that SLs and CKs act antagonistically on bud outgrowth in garden pea (Dun et al., 2012). Recently, SLs have been shown to promote CK degradation through the transcriptional activation of *CYTOKININ OXIDASE/DEHYDROGENASE 9* in rice (Duan et al., 2019). However, it is not clear if CKs act on shoot branching by affecting SL production or not. The involvement of CKs in the repression of Pi starvation signaling has been well documented, and it was reported that CK application down-regulates the expression of several Pi starvation-responsive genes (Martín et al., 2000; Wang et al., 2006).

In this study, to clarify if Pi and CK interact to regulate SL biosynthesis or if they act independently, the effects of CK treatment at different concentrations on SL production and exudation were examined in hydroponically grown rice plants. In addition, the expression of genes involved in SL biosynthesis and signal transduction was quantified in rice roots treated with CK. Furthermore, Pi levels were determined in the plants treated with CK. The endogenous *cis*-zeatin levels were compared between the plants grown under Pi deficiency and those subjected to Pi fertilization.

## Results

### Cytokinins Inhibited 4-Deoxyorobanchol Production and Exudation

Although it was reported that both N and Pi deficiency increased the levels of 4DO, one of the major SLs in rice plants, in root tissues and root exudates (Shindo et al., 2018), only Pi starvation but not N starvation increased the 4DO levels in rice (*Oryza sativa* cv. Nipponbare) root tissues under our experimental conditions (Supplementary Figure S1). Therefore, rice plants were grown hydroponically under Pi deficiency, and then each CK, including *t*-zeatin, benzyladenine, and CPPU, was applied to the hydroponic culture media. Plants grown under Pi-fertilized conditions were used as a negative control in which the suppression of 4DO production and exudation occurred. 4DO was quantified by LC-MS/MS (MRM, multiple reaction monitoring).

**FIGURE 2:**
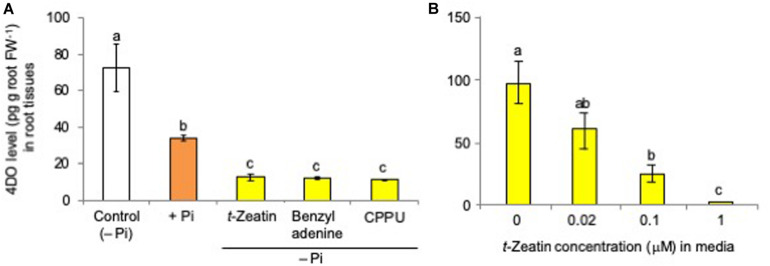
**(A)** Cytokinin application suppressed the 4-deoxyorobanchol (4DO) levels in root tissues. **(B)** The 4DO levels in root tissues decreased with the increase of *t*-zeatin concentrations in culture media. The rice plants were grown under phosphate (Pi) deficiency for 10 days, and then 10 plants each were transferred to Pi-depleted media containing 1 μM cytokinin. Control was continued with Pi-deficient conditions. At 24 h after treatment, the root contents of 4DO were quantified by LC-MS/MS (MRM). Data are means ± SE (*n* = 3). Different letters indicate statistically significant differences according to Tukey’s honestly significant difference test (*P* < 0.05).

As in the case of Pi fertilization, CKs (final concentration, 1 μM) significantly suppressed the 4DO levels in root tissues (Figure 2A) and root exudates of rice plants (Supplementary Figure S2A), even those grown under Pi-deficient conditions. CPPU [*N*-(2-chloro-4-pyridyl)-*N’*-phenylurea], a phenylurea-type CK which is structurally distinct from adenine-type CKs, also suppressed 4DO levels, suggesting that this inhibitory effect on 4DO production is attributable to CK activity. The suppression of 4DO production and the exudation by CK application were also observed in the SL perception and signaling mutant *d3* (Supplementary Figure S3). This inhibitory effect of CK on 4DO production and exudation is completely and inversely dependent on CK concentrations in the culture media. The 4DO levels in both root tissues (Figure 2B) and root exudates (Supplementary Figure S2B) decreased with the increase of *t*-zeatin concentrations in the culture media. The negative effect of CK (*t*-zeatin and CPPU) application also occurred in dicotyledonous tomato plants (Supplementary Figure S4).

**FIGURE 3:**
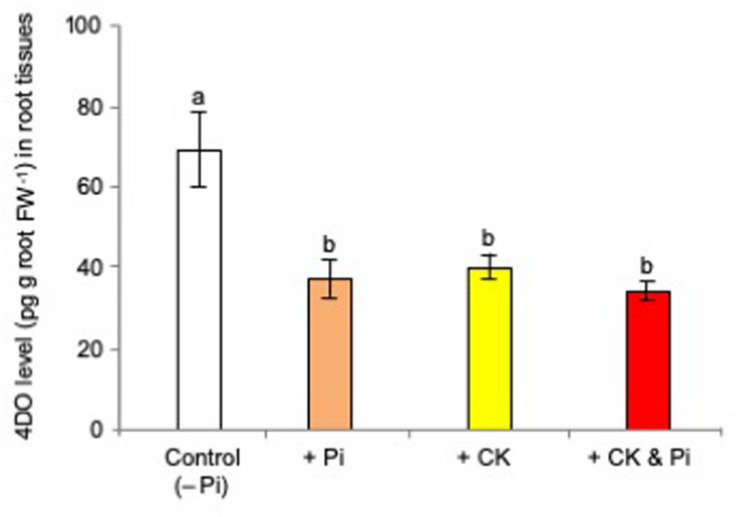
Simultaneous application of cytokinin (*t*-zeatin) and phosphate (Pi) did not reduce the 4-deoxyorobanchol (4DO) contents further. The rice plants were grown under Pi deficiency for 10 days, and then 10 plants were used for each treatment with three replicates. The concentrations of *t*-zeatin and Pi were set to 0.05 μM and 20 mM, respectively, at which 4DO production was reduced to 60–70%. At 24 h after treatment, the root contents of 4DO were quantified. Data are means ± SE (*n* = 3). Different letters indicate statistically significant differences according to Tukey’s honestly significant difference test (*P* < 0.05).

Then, the effects of the simultaneous application of *t*-zeatin and Pi on 4DO production and exudation were examined by treating 0.05 μM *t*-zeatin and 20 μM Pi at which 4DO production was reduced to 60–70% *versus* that of the untreated controls. The simultaneous application of *t*-zeatin and Pi did not reduce the 4DO root contents further (Figure 3).

### Cytokinin Only Locally Inhibited SL Production

**FIGURE 4:**
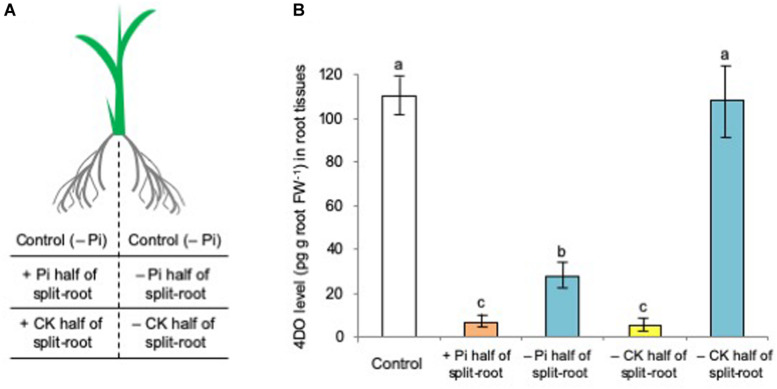
Phosphate (Pi) systemically and cytokinin (CK, *t*-zeatin) locally suppressed the 4-deoxyorobanchol (4DO) levels in root tissues. **(A)** Illustration of a split-root plant. **(B)** The plants were grown under phosphate (Pi) deficiency for 14 days and then subjected to CK treatment (1 μM *t*-zeatin) and Pi fertilization (160 mM Pi) to only half of the split-root. At 24 h after treatment, root exudations of 4DO were quantified. Data are means ± SE (*n* = 3). Different letters indicate statistically significant differences according to Tukey’s honestly significant difference test (*P* < 0.05).

Our previous study in a split-root system (Figure 4A) of sorghum plants demonstrated that the SL levels significantly decreased not only in the Pi-fertilized roots but also in the non-fertilized roots (Yoneyama et al., 2015). Phosphate was not translocated from the fertilized roots into the non-fertilized roots, indicating that Pi systemically regulates SL production and exudation. Then, the effects of *t*-zeatin application and Pi fertilization on SL production were examined by using the same split-root system in rice plants. As expected, the 4DO levels were reduced not only in the Pi-fertilized roots but also in the non-fertilized roots (Figure 4B). In contrast, a significant reduction of 4DO level could be observed only in the roots applied with *t*-zeatin but not in the roots not applied with *t*-zeatin (Figure 4B).

### Cytokinin Application as Well as Pi Fertilization Suppressed the Expression of SL Biosynthesis Genes

**FIGURE 5:**
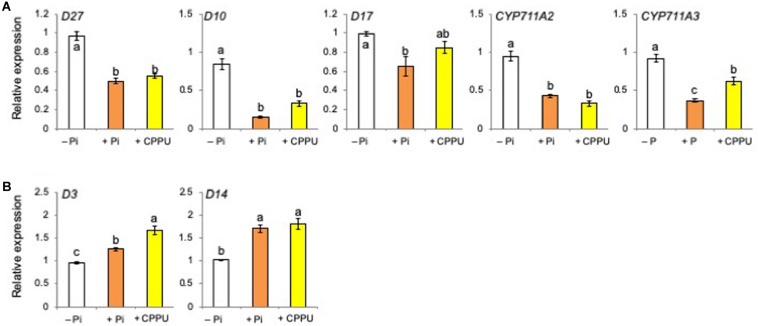
Both cytokinin (CPPU) application and phosphate (Pi) fertilization decreased the expression of strigolactone (SL) biosynthetic genes **(A)** while increasing SL perception and signaling genes **(B)**. The plants were grown under Pi deficiency for 14 days and then subjected to CPPU treatment (1 μM) and Pi fertilization (160 mM Pi). RNA was extracted from the root tissues. Expression is represented relative to the –Pi treatment; UBQ was used as the internal reference gene. Data are means ± SE (*n* = 3). Different letters indicate statistically significant differences according to Tukey’s honestly significant difference test (*P* < 0.05).

Total RNA was extracted from the root tissues of rice plants grown under Pi deficiency, Pi fertilized, and CK (CPPU) applied conditions, and then the relative expression levels of SL biosynthesis and perception genes were quantified by real-time PCR. Umehara et al. (2008) reported that Pi fertilization to pre-incubated Pi-starved plants suppressed the expression of SL biosynthetic genes. Similar results were obtained in the present study, and not only Pi fertilization but also CK (CPPU) application decreased the expression of SL biosynthetic genes (Figure 5A), while the expression of SL perception and signaling genes increased (Figure 5B). These results suggest that CK as well as Pi fertilization suppresses SL biosynthesis.

### Phosphate Fertilization Affected CK Biosynthesis

It is important to clarify if CK represses Pi starvation responses, including SL production, or if Pi fertilization promotes CK biosynthesis which then suppresses SL production.

The involvement of CKs in the repression of Pi starvation signaling has been well documented, and it was reported that CK application down-regulates the expression of several Pi starvation-responsive genes (Martín et al., 2000; Wang et al., 2006). Such a negative regulation may be attributable in part to the CK-induced release of inorganic phosphate from internal sources. The subsequent elevation of intracellular Pi levels in plant tissues down-regulates Pi starvation-responsive genes (Wang et al., 2006). Therefore, inorganic P (Pi) levels were determined and compared between Pi-fertilized and CK (CPPU)-applied rice plants. However, in this study, CK (CPPU) application did not affect the Pi levels in root tissues (Supplementary Figure S5).

**FIGURE 6:**
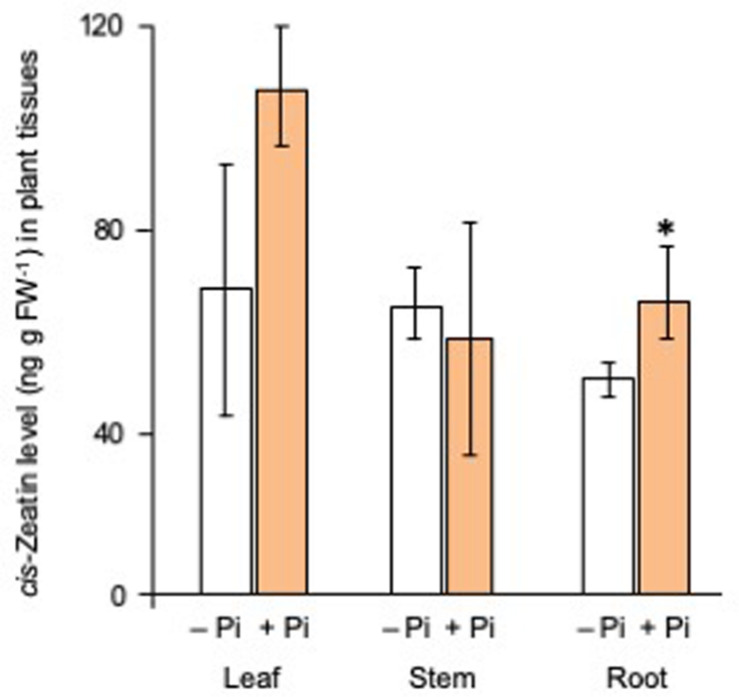
Phosphate (Pi) fertilization increased the *cis*-zeatin levels in root tissues. The plants were grown under Pi deficiency for 14 days, and then 10 seedlings each were subjected to Pi fertilization (160 mM Pi). *cis*-Zeatin was extracted from each tissue and quantified by LC-MS/MS. Data are means ± SE (*n* = 3). Asterisks indicate significant differences according to Student’s *t-*test (*P* < 0.05).

**FIGURE 7:**
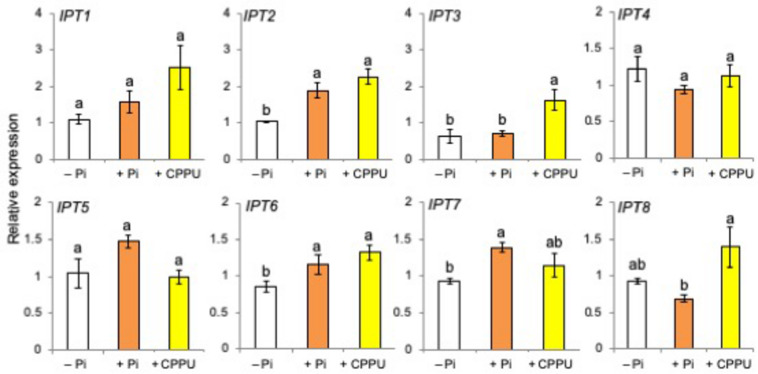
The expression of cytokinin biosynthetic genes including *IPT2*, *IPT6*, and *IPT7* increased by both phosphate (Pi) fertilization and CPPU application. The plants were grown under Pi deficiency for 14 days and then subjected to CPPU treatment (1 μM) and Pi fertilization (160 mM Pi). RNA was extracted from the root tissues. Expression is represented relative to the –Pi treatment; UBQ was used as the internal reference gene. Data are means ± SE (*n* = 3). Different letters indicate statistically significant differences according to Tukey’s honestly significant difference test (*P* < 0.05).

Then, the levels of *cis*-zeatin, a major active CK of rice plants, in the leaf, the stem, and the root tissues were compared between Pi-deficient and Pi-fertilized conditions. Phosphate fertilization increased the levels of *cis*-zeatin in root tissues (Figure 6). As shown in Figure 7, the expression of CK biosynthetic genes, including *IPT2*, *IPT6*, and *IPT7*, increased by both Pi fertilization and CK (CPPU) application, indicating that Pi fertilization and CK application may promote CK biosynthesis. The expression of all type-A response regulator genes tended to increase by CK (CPPU) application, but the differences were not statistically significant (Supplementary Figure S6).

## Discussion

SLs play pivotal roles as multifunctional plant hormones, and SL production and exudation are precisely regulated through crosstalk with other plant hormones. In the present study, we demonstrated that CK, a canonical plant hormone known as a shoot-branching promoter, is a negative regulator for SL production and exudation in rice plants. Exogenous *t*-zeatin and CPPU decreased the SL levels also in tomato plants (Supplementary Figure S4), suggesting that elevated CK levels in plants negatively regulate SL production and exudation not only in rice but also in other plant species.

Then, a question arises as to whether CK and Pi independently retard SL production and exudation or they act on the same step in SL production. Because the simultaneous application of CK (CPPU) and Pi did not cause a further reduction of SL levels, Pi and CK appear to regulate the same step in SL biosynthesis (Figure 3). Both CK and Pi downregulated SL biosynthetic genes (Figure 5A) and upregulated SL perception and signaling genes (Figure 5B). A distinct difference between CK and Pi is that Pi systemically but CK only locally suppressed SL production (Figure 4). Although CK is mobile from roots to shoots and suggested to function as a systemic signal in the response to nutrient availability (Takei et al., 2001), not CK but Pi appears to function as a systemic signal for SL production. The fact that CK can suppress SL production locally while Pi affects SL production systemically suggests that Pi and CK act on SL production independently. Then, it was found that Pi fertilization upregulated the gene expression of several *IPT*s (Figure 7) and increased the *cis*-zeatin levels in root tissues (Figure 6). In *Arabidopsis*, *AtIPT3* is a key gene for limiting step in the biosynthesis of CKs (Krouk et al., 2011), and its expression was induced by Pi application (Hirose et al., 2008). However, the gene expression of rice *IPT3* under our experimental conditions was not influenced by Pi fertilization (Figure 7).

In contrast to CK, auxin is a positive regulator of SL biosynthesis; auxin upregulates SL biosynthetic genes (Foo et al., 2005) and promotes SL production (Yoneyama et al., 2015). Although IAA application increased 5DS, one of the major SLs produced by sorghum plants, under Pi deficiency, IAA was not effective under Pi-sufficient conditions (Yoneyama et al., 2015). These results suggest that IAA and Pi act somewhat independently to regulate SL production or at least high Pi is epistatic to high IAA.

Gibberellins (GAs) have been suggested to affect SL production and exudation (Ito et al., 2017). The regulation of SL biosynthesis by GAs is dependent on the GA receptor GID1, and application of GA reduced the SL levels in roots and root exudates. However, GAs and SLs seem to regulate separate processes (Bennett et al., 2016) and function independently in stem elongation (de Saint Germain et al., 2013; Lantzouni et al., 2017). Abscisic acid (ABA) is another plant hormone derived from carotenoids. Since mutants impaired in ABA biosynthesis produce lower levels of SLs, ABA has been suggested to regulate SL biosynthesis (López-Ráez et al., 2010; Torres-Vera et al., 2014). Some direct and indirect interactions between ABA and SLs seem to occur, but no clear evidence has been reported so far. Other plant hormones, brassinosteroids, jasmonic acid, salicylic acid, and ethylene, may also interact with SLs, while further studies are needed to draw a clear conclusion.

SLs have been shown to participate directly in adaptive responses to abiotic stresses such as drought and salinity in *Arabidopsis* (Ha et al., 2014). Although various plant hormone biosynthesis and signaling mutants of *Arabidopsis* are available, SL production was not promoted by Pi deficiency in *Arabidopsis* (Seto et al., 2014). Therefore, it is difficult to clarify the effects of complex interactions among plant hormones and Pi on SL production in this model plant. Plant hormone levels at various growth and developmental stages may differently influence SL production and exudation, which in turn influences the physiological functions of SLs such as the inhibition of shoot branching.

## Materials and Methods

### Plant Material

Rice seeds (*O. sativa* cv. Nipponbare) were obtained from a local supplier. Seeds of rice *d3-2* mutant (Nipponbare background, Yoshida et al., 2012) were a generous gift from Prof. Junko Kyozuka (Tohoku University).

### Chemicals

4-Deoxyorobanchol and 3a,4,4,5,5,6’[^2^H_6_] 4-deoxyorobanchol were generous gifts from Prof. Kohki Akiyama (Osaka Prefecture University) and Prof. Tadao Asami (The University of Tokyo), respectively. [^2^H_5_] Zeatin, trans isomer, was obtained from OIChemIm Ltd. (Czech Republic). The other chemicals of analytical grade and the HPLC solvents were obtained from Kanto Chemical Co., Ltd. and Wako Pure Chemical Industries Ltd.

### Hydroponic Culture of Rice Plants

Rice seeds were surface-sterilized in 70% ethanol for 3 min and thoroughly rinsed with sterile Milli-Q water. Then, the seeds were germinated on moistened filer paper in Petri dishes for 2 days at 30°C in the dark. The seedlings (*n* = 10) were transferred to a stainless steel sieve lined with a sheet of gauze which was moistened by placing it on a plastic cup (9.5 cm in diameter, 17 cm deep, ca. 550 ml in volume) containing 500 ml of tap water. The plants were grown in a growth chamber with 14/10 h photoperiod at 120 μmol photons m^–2^ s^–1^ at 30/28°C. Half-strength Tadano and Tanaka (TT) medium (Tadano and Tanaka, 1980), containing 2.43 mM N, 0.16 mM P, 1 mM K, 1 mM Ca, and 1 mM Mg, was used as the basal culture medium with 1 mM 4-morpholineethanesulfonic acid. The pH of all culture media was adjusted to 6.0 with KOH. The seedlings were grown hydroponically with tap water for 7 days and in 1/2 TT medium for another 7 days. Then, the plants were subjected to each nutrient condition for 14 days. Control means 1/2 of TT media. The respective N-deficient and Pi-deficient solutions were prepared by respectively removing each element from the 1/2 TT media. Split-root system was conducted as reported previously (Yoneyama et al., 2015).

Tomato plants (10 plants per replicate) were grown in a similar manner: 6 days in tap water, 6 days in 1/2 TT medium, and then 14 days under Pi-deficient conditions.

### Cytokinin Application

Authentic standards of CKs were dissolved in dimethyl sulfoxide (DMSO), and only DMSO was applied to control and negative control (Pi fertilization) conditions.

### Extraction of SLs From Root Exudates

SLs were extracted from root exudates and tissues as reported previously (Yoneyama et al., 2012, 2015). Briefly, the root exudates from 10 plants were collected and extracted with ethyl acetate. The ethyl acetate solutions were washed with 0.2 M K_2_HPO_4_, dried over anhydrous MgSO_4_, and concentrated *in vacuo*. The harvested root tissues were soaked in ethyl acetate in the dark at 4°C for 2 days, and then filtrated, washed with 0.2 M K_2_HPO_4_, dried over anhydrous MgSO_4_, and concentrated *in vacuo*. These samples were kept at 4°C until analysis.

### Extraction of CKs From Root Tissues

CKs were extracted from the plant tissues of 10 plants as reported previously (Kojima et al., 2009). Briefly, the powder of root tissues ground with liquid nitrogen (ca. 500 mg) was mixed vigorously in 80% acetonitrile containing 1% acetic acid and an internal standard of *t*-zeatin-*d*_5_ (1 ng) and kept at –20°C overnight. After centrifugation at 14,000 × *g* for 10 min, the supernatant was collected. Crude extracts were purified using a C_18_ column. The samples were kept at 4°C until analysis.

### Quantification of SLs and CKs

SL and CK were analyzed by LC-MS/MS as reported previously (Kojima et al., 2009; Abe et al., 2014). Briefly, LC-MS/MS analysis (MRM and PIS) of proton adduct ions was performed with a triple quadruple/linear ion trap instrument (QTRAP5500; AB Sciex, Old Connecticut Path Framingham, MA, United States) with an electrospray source. HPLC separation was performed on a UHPLC (Nexera X2; Shimadzu) equipped with an ODS column (Kinetex C_18_, 2.1 × 150 mm, 1.7 μm; Phenomenex) with a linear gradient of 35% (0 min) to 95% acetonitrile (20 min) for SL and a gradient of 3% (0 min) to 25% acetonitrile (27 min) for CK. The column oven temperature was maintained at 30°C.

### Quantification of Gene Expression

Total RNA was extracted from the roots of 10 plants using RNeasy Plant Mini Kit (QIAGEN). cDNA was synthesized with the PrimeScript RT Reagent Kit with gDNA eraser (Takara Bio). qRT-PCR was performed on a StepOnePlus real-time PCR system (Applied Biosystems) with FastStart Essential DNA Green Master. The specific primers used for qRT-PCR are listed in Supplementary Table S1.

### Quantification of Inorganic Phosphate

Powder of the root tissues of 10 plants ground with liquid nitrogen (ca. 500 mg) was mixed vigorously in 10% (w/v) perchloric acid and placed on ice for 30 min. After centrifugation at 10,000 × *g* for 10 min at 4°C, the supernatant was used to measure Pi using the molybdate-blue method (Nanamori et al., 2004).

### Statistical Analysis

All experiments with three replicates were repeated at least twice to confirm the results. The data presented are mean ± standard error (*n* = 3) from a typical single experiment. The results were analyzed by analysis of variance (JMP 7, SAS institute Inc., Cary, NC, United States) followed by Tukey’s honestly significant difference and Student’s *t*-tests.

## Data Availability Statement

The datasets generated for this study are available on request to the corresponding author.

## Author Contributions

KaY, XX, TN, and KoY designed the experiments. KaY performed the experiments. KaY and KoY wrote the manuscript. All the authors contributed to the discussion and approved the final manuscript.

## Conflict of Interest

The authors declare that the research was conducted in the absence of any commercial or financial relationships that could be construed as a potential conflict of interest.

## Supplementary Material

FIGURE S1 Effects of nitrogen and phosphate on 4-deoxyorobanchol levels in the root tissues of rice plants (cv. Nipponbare). Different letters indicate statistically significant differences according to Tukey’s HSD test (*P* < 0.05).

FIGURE S2 Effects of *t*-zeatin, benzyladenine, and CPPU on 4-deoxyorobanchol levels in the rice root exudates. Different letters indicate statistically significant differences according to Tukey’s HSD test (*P* < 0.05).

FIGURE S3 Effects of *t*-zeatin on 4-deoxyorobanchol levels in the root tissues of rice *d3* mutant. Different letters indicate statistically significant differences according to Tukey’s HSD test (*P* < 0.05).

FIGURE S4 Effects of phosphate fertilization, *t*-zeatin, and CPPU on orobanchol levels in the root tissues of tomato plants. Different letters indicate statistically significant differences according to Tukey’s HSD test (*P* < 0.05).

FIGURE S5 Effects of CPPU on inorganic phosphate status. Different letters indicate statistically significant differences according to Tukey’s HSD test (*P* < 0.05).

FIGURE S6 Effects of phosphate fertilization and CPPU on gene expression of the type-A response regulator. Different letters indicate statistically significant differences according to Tukey’s HSD test (*P* < 0.05).

TABLE S1 Primer sequences used for qRT-PCR.
