# Supplementary figures and images for: Do Phosphate and Cytokinin Interact to Regulate Strigolactone Biosynthesis or Act Independently?
Source: Front Plant Sci. 2020 May 20;11:438. doi: 10.3389/fpls.2020.00438 (PMC7251057; doi:10.3389/fpls.2020.00438)

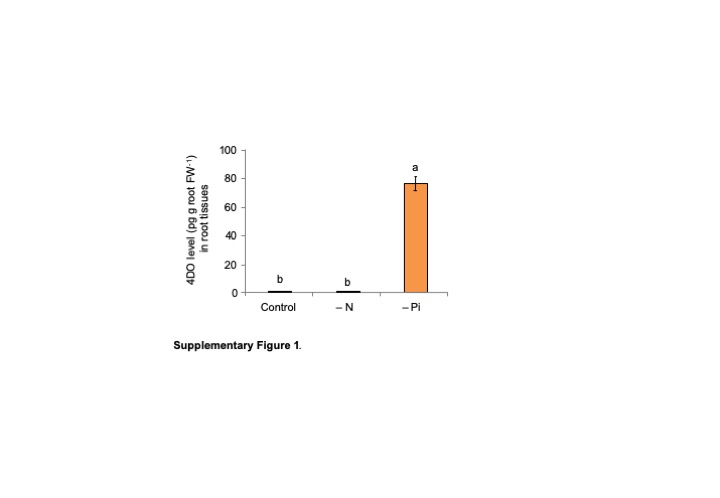

Supplement: FIGURE S1 — Effects of nitrogen and phosphate on 4-deoxyorobanchol levels in the root tissues of rice plants (cv. Nipponbare). Different letters indicate statistically significant differences according to Tukey’s HSD test (P < 0.05). [file Image_-1.jpeg]

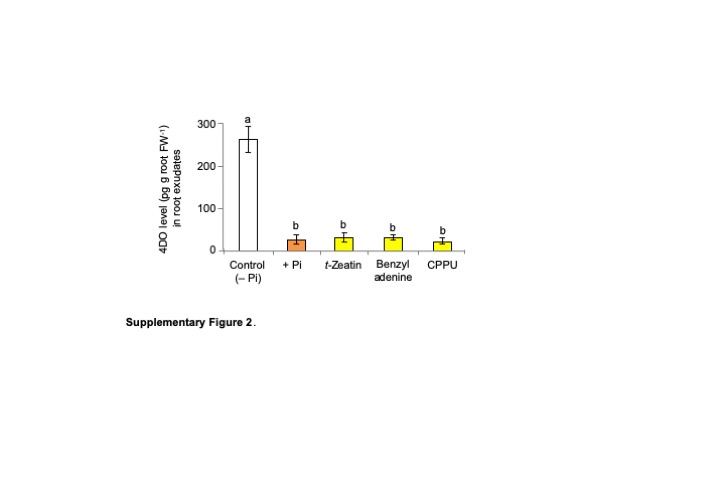

Supplement: FIGURE S2 — Effects of t-zeatin, benzyladenine, and CPPU on 4-deoxyorobanchol levels in the rice root exudates. Different letters indicate statistically significant differences according to Tukey’s HSD test (P < 0.05). [file Image_2.jpeg]

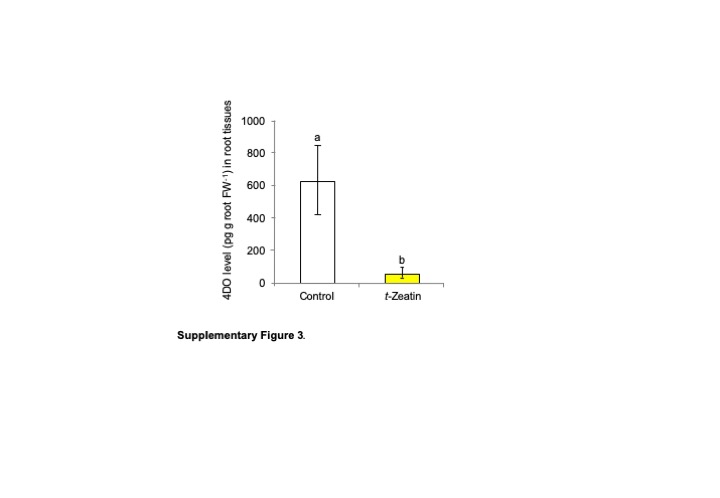

Supplement: FIGURE S3 — Effects of t-zeatin on 4-deoxyorobanchol levels in the root tissues of rice d3 mutant. Different letters indicate statistically significant differences according to Tukey’s HSD test (P < 0.05). [file Image_3.jpeg]

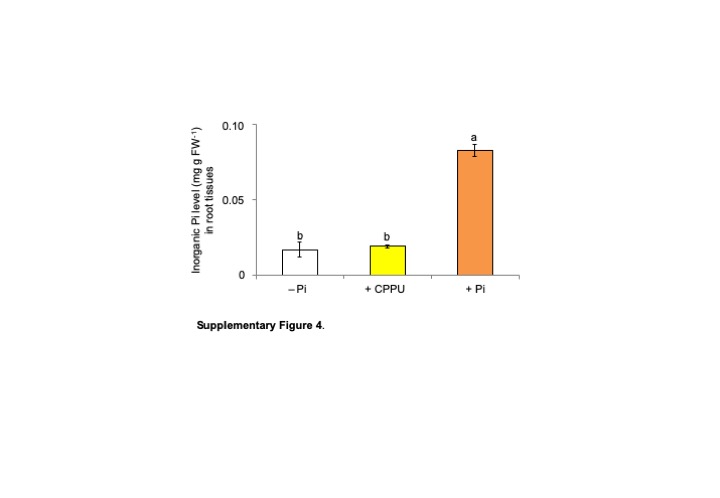

Supplement: FIGURE S4 — Effects of phosphate fertilization, t-zeatin, and CPPU on orobanchol levels in the root tissues of tomato plants. Different letters indicate statistically significant differences according to Tukey’s HSD test (P < 0.05). [file Image_4.jpeg]

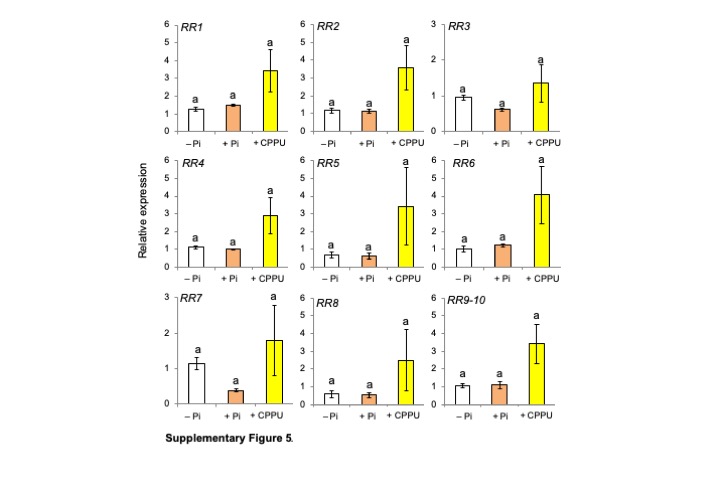

Supplement: FIGURE S5 — Effects of CPPU on inorganic phosphate status. Different letters indicate statistically significant differences according to Tukey’s HSD test (P < 0.05). [file Image_5.jpeg]

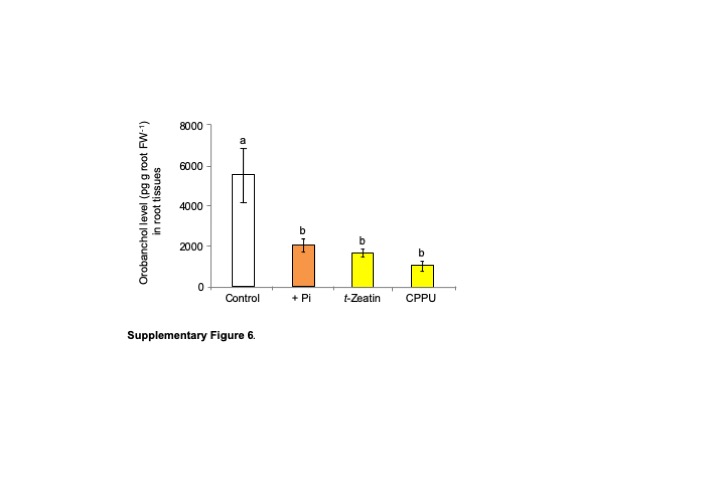

Supplement: FIGURE S6 — Effects of phosphate fertilization and CPPU on gene expression of the type-A response regulator. Different letters indicate statistically significant differences according to Tukey’s HSD test (P < 0.05). [file Image_6.jpeg]
