# Supplementary material for: Do Phosphate and Cytokinin Interact to Regulate Strigolactone Biosynthesis or Act Independently?
Source: Front Plant Sci. 2020 May 20;11:438. doi: 10.3389/fpls.2020.00438 (PMC7251057; doi:10.3389/fpls.2020.00438)
Supplement: TABLE S1 — Primer sequences used for qRT-PCR. [file Table_1.pdf]

Table S1. Primer sequences used for qRT-PCR

Primer name	5' to 3' sequence	Primer name	5' to 3' sequence
D27-F	AGATGACCCTGCATTGAAGCA	*RR1-F*	AGGATCAGCAGATGCATGAATG
D27-R	GCAATTCACACCATGTTCTGC	*RR1-R*	GAGACGCTGTACGTCCTTGCTT
D10-F	CTGTACAAGTTCGAGTGGCACC	*RR2-F*	ACGATCTTCTCAAAGCCATCAAG
D10-R	CCTGTCCGTCTCCTCGTAC	*RR2-R*	TGAGAGGCTTAAGGATGAAATCCT
D17-F	CCTCGTCCAGAAGCGTGAG	*RR3-F*	CAGGGTTCGATCTCCTCAAGAG
D17-R	TAGTGGGTGTCGGTGAAGGC	*RR3-R*	CGAATTCTCCGACGACATTAGC
CYP711A2-F	CGTGAACCTCACGCTCGG	*RR4-F*	TCTTCTGAGAATGTGCCTGCAA
CYP711A2-R	TTCATTGCAGCCGTCCG	*RR4-R*	GCTTGACAGGTTTCAGGAAGAACT
CYP711A3-F	TGCATTGAGTGCGTGTCCA	*RR5-F*	ACCGAATGTGAGCATGATTATCA
CYP711A3-R	GAAGCCGAGAGCGAGATCG	*RR5-R*	CCTTGACCTTCTTCAGGAGTTCATA
D3-F	CCCAACCTCCGCAAGCT	*RR6-F*	GTCCCCAACGTCAACATGATC
D3-R	GACGCAATCGCTGAACCG	*RR6-R*	CACGTTCTCCGACGACATGAT
D14-F	GCCTCTCCCCGGTTCTTG	*RR7-F*	TGCTCAAGAAGATCAAGGAATCG
D14-R	TGCTGTATCTCCTCCAGCTCG	*RR7-R*	GGCACGTTCTCTGACGACATTAT
IPT1-F	ACCAAGCCCAAGGTTATCTTCGTGC	*RR8-F*	CCAGACATGACCGGCTATAACC
IPT1-R	TCGTCGGTGACCTTGTTGGTGATGA	*RR8-R*	AAGCAATTACAACCGGGAGATG
IPT2-F	AGTCACCCCCCCCCAAGCCCAAGGTCGTCTT	*RR9-10-F*	TCATGAGGACAGCCCAATTTCTA
IPT2-R	CTCCTCGGTGACCTTGTTCGTGATG	*RR9-10-R*	TGCAGTAGTCTGTGATGATCAGGTT
IPT3-F	GAGCTGTGCTTCCTGTGGGTGGACT	*Ubiquitin-F*	AAGGTCACCAGGCTCAGGAAG
IPT3-R	GCGACCTTGTACTTGTCTCCGTGCG	*Ubiquitin-R*	GATCGAAGTGGTTGGCCATG
IPT4-F	TGGATGTGGTGACGAACAAGGTGAC		
IPT4-R	GATCTACGTCGACCCAGAGGAAGCA		
IPT5-F	AGGTGATCAACGCCGACAAGCTGCA		
IPT5-R	TCGACGAGCTCCTCGATGTAGGACT		
IPT6-F	GATCGATGCGGCATATCTCATCACC		
IPT6-R	CCTCCAATTGCCCAAAAGGATCCAC		
IPT7-F	TGGACGACATGGTGGACGCTGGCAT		
IPT7-R	GCTTTGATGTCGTCGATCGCCTCGG		
IPT8-F	GTCGACGACGATGTTCTCGACGAAT		
IPT8-R	TGTTGGCCTTGATCTCGTCTATCGC		
